# Abnormal Default-Mode Network Homogeneity in Patients With Mild Cognitive Impairment in Chinese Communities

**DOI:** 10.3389/fneur.2020.569806

**Published:** 2021-02-11

**Authors:** Yuping Cao, Huan Yang, Zhenhe Zhou, Zaohuo Cheng, Xingfu Zhao

**Affiliations:** ^1^Mental Health Institute, The Second Xiangya Hospital, Central South University, Changsha, China; ^2^China National Clinical Research Center on Mental Disorders, Changsha, China; ^3^China National Technology Institute on Mental Disorders, Changsha, China; ^4^Hunan Technology Institute of Psychiatry, Changsha, China; ^5^Hunan Key Laboratory of Psychiatry and Mental Health, Changsha, China; ^6^Wuxi Mental Health Center, Nanjing Medical University, Wuxi, China

**Keywords:** mild cognitive impairment, cognitive problems, default-mode network, network homogeneity, rest-fMRI

## Abstract

**Background and Objective:** Current evidence suggests that abnormalities within the default-mode network (DMN) play a key role in the broad-scale cognitive problems that characterize mild cognitive impairment (MCI). However, little is known about the alterations of DMN network homogeneity (NH) in MCI.

**Methods:** Resting-state functional magnetic resonance imaging scans (rs-fMRI) were collected from 38 MCI patients and 69 healthy controls matched for age, gender, and education. NH approach was employed to analyze the imaging dataset. Cognitive performance was measured with the Chinese version of Alzheimer's disease assessment scale-Cognitive subscale (ADAS-Cog).

**Results:** Two groups have no significant differences between demographic factors. And mean ADAS-Cog score in MCI was 12.02. MCI patients had significantly lower NH values than controls in the right anterior cingulate cortex and significantly higher NH values in the ventral medial prefrontal cortex(vmPFC) than those in healthy controls. No significant correlations were found between abnormal NH values and ADAS-Cog in the patients.

**Conclusions:** These findings provide further evidence that abnormal NH of the DMN exists in MCI, and highlight the significance of DMN in the pathophysiology of cognitive problems occurring in MCI.

## Introduction

Mild cognitive impairment (MCI) is recognized as cognitive decline more significant than the expectation of an individual's age and education level (1). Still, it does not obviously interfere with individuals' daily activities. MCI demonstrates as an intermediate state between age-related cognitive decline and dementia (2). The prevalence of MCI among people aged 65 years or older in China is 14.7–20.8% (3). Among people with MCI, approximately one third to half of them have an accelerated rate of progression to dementia within 5 years (1, 4). Recent researches demonstrated that people who suffer MCI tended to develop to AD at a rate of about 10–15% per year (5) compared with the ordinary aged people who grow to AD at a rate of 1–2% per year (6). MCI can thus regard as a prodromal stage and a risk state for dementia. Its early identification could provide secondary prevention by controlling risk factors, such as systolic hypertension (1), and can also promote early interventions to improve cognitive function, such as cognitive training (7).

The default mode network (DMN) was first reported by Raichle et al. to explicate decreased activation in neuroimaging studies of attention-demanding cognitive tasks (8). During demanding tasks in MCI individuals, failure to deactivate this network sufficiently may imply impairments in goal-directed attention and working memory (9–11). Moreover, the DMN is essential in cognitive functions, including future thinking, planning, spatial navigation, and conceptual processing that impaired in MCI (12, 13).

Many studies exhibited evidence for the role of the DMN in age-related cognitive impairment (14–16). Not limited to AD, other age-related disorders with cognitive impairment also demonstrated DMN alterations, such as vascular cognitive impairment (17) and Parkinson's disease (18). Functional magnetic resonance imaging (fMRI) is valuable for the identification of cognitive impairment biomarkers (5, 6). Measuring functional connectivity (FC) of the resting state is a method of analyzing functional networks without stimuli from the external environment (19). Evidence demonstrated abnormal resting-state FC in the DMN of MCI. Functional connectivity within the DMN tends to decline with healthy aging but demonstrated accelerated decreases in AD (20, 21). However, in previous studies, connectivity methods were used to analyze abnormal FC, for example, seed-based (22, 23) and seed-free voxel-wise connectivity methods (24, 25). To date, seed-based resting-state fMRI methods had been used by numerous studies to examine DMN connectivity in MCI, reporting altered connectivity within the DMN and between DMN regions and task-positive network regions (26–28). A limitation of seed-based methods is that they demand that seeds need to be chosen at first. Other researches have used relatively less biased seed-free voxel-wise connectivity methods (29, 30). However, these methods also have limitations as it is unclear whether intra-network or inter-network connections drive findings for a particular region. For these reasons, using the DMN network homogeneity (NH) metric to study DMN functioning in MCI might be a good way. This method uses each voxel within a DMN mask as a seed to determine its mean connectivity to all other DMN voxels (31). It therefore provides an unbiased survey of DMN intra-network connectivity without the need for a priori knowledge of where in the network abnormalities might exist.

In our research, NH was used to explore MCI patients' functional connectivity in DMN. Based upon previous FC findings in the DMN among MCI patients, it was hypothesized that MCI individuals would show abnormal homogeneity within the DMN compared with age-matched controls (32). We also explored whether abnormal NH in the DMN would be associated with patient neurocognitive functioning and in clinical variables associated with MCI.

## Materials and Methods

### Subjects

All subjects were recruited from four neighborhood committees in Wuxi City (2). With the assistance of community staff, a questionnaire (self-designed) was conducted among community residents over 50 years via household surveys administered by medical personnel from social prevention and control departments of Wuxi Mental Health Center. The contents of the study included the willingness to join in this project, general conditions (e.g., name, age, and education level), and medical history, the latter of which focused on whether or not individuals had illnesses in the exclusion criteria.

The Chinese version of Alzheimer's disease assessment scale-Cognitive subscale (ADAS-Cog) was used to assess cognitive function in patients. It has been proved that the total score of ADAS-Cog can detect cognitive impairment of AD patients in Chinese communities (2).

#### Inclusion Criteria

Both patients and controls need to be aged ≥50 years and voluntarily accepted ADAS-Cog and signed relevant informed consent. All patients having minimum reading and writing ability (able to fill out the questionnaire independently, regardless of whether or not have received formal education) presented with mild cognitive impairment, as indicated by scores that were ≥10 on ADAS-Cog. Controls without reading and writing ability problems need to score lower than 10 on ADAS-Cog.

#### Exclusion Criteria

Individuals with any disease that may cause cognitive decline or dementia will be excluded from the study, such as cerebrovascular disorder, neurodegenerative disorders, head trauma, neurosyphilis, and vital organ failure. Furthermore, subjects with major mental health disorders, including depression, schizophrenia, and other psychiatric illnesses, substance abuse, severe metabolic disturbances impacting mental function, or any systemic diseases resulting in hypoxia were excluded.

The Medical Ethics Committee of Wuxi Mental Health Center approved the study. All of the participants or their legal representatives signed written informed consent, which included the purposes of the research and the confidentiality of the provided information.

### Scan Acquisition

Scanning was conducted on the GE 3.0T MR scanner. Brain structural MRI data were collected with a three-dimensional (3D) magnetization-prepared rapid gradient echo (MP-RAGE) squeeze with the following parameters: repetition time (TR) = 7.7 s; echo time (TE) with the minimum value; field of view (FOV), 256 × 256 mm; acquisition matrix, 256 × 256; flip angle (FA), 11°; and slice thickness, 1.2 mm with 186 slices. Data of rs-fMRI were collected while participants lie down with eyes closed but remain awake. No participants reported falling asleep during the scanning session. We used a prototype quadrature birdcage head coil fitted with foam padding to minimize the head movement. Functional data were collected using the following parameters: repetition time (TR), 2,000 ms; time to echo, 30 ms; time for inversion, 100 ms; flip angle (FA), 90°; field of view, 224 × 224 mm; matrix size, 64 × 64; slice thickness, 3.5 mm; voxel size, 3.5 × 3.5 × 3.5 mm^3^; and 240 volumes in total. None of the participants had structural abnormalities upon visual inspection of the scans.

### Data Preprocessing

Preprocessing rs-fMRI imaging data was done by Data Processing Assistant for Resting-State fMRI software (DPARSF; http://rfmri.org/DPARSF) in MATLAB (Mathworks) (33). First, the first five time points were removed. Then, slice time and head motion correction were accomplished. No participants had been dropped during the steps of preprocessing. Subsequently, normalization and resampling were applied to generate the dimensions of 3 × 3 × 3 mm. Also, a voxel size of 3 × 3 × 3 mm was used as the functional covariate. Temporal scrubbing using motion “spikes” [framewise displacement (FD) > 1] as separate repressors were performed. The scrubbing effectively censored the data at the spike without further changing the correlation values. After that, an 8-mm full-width at half-maximum Gaussian kernel was used to smooth the acquired images. Temporal bandpass filtering (0.01–0.08 Hz) and linear detrending were applied to lessen the influence of low-frequency drifts and high-frequency physiological noise. During the preprocessing, the signal from a region centered in the white matter, six head motion parameters generated by rigid body correction, and the signal from a ventricular region of interest were removed. However, on consideration of removing the global signal may introduce artifacts into the data and distort resting-state connectivity patterns and the regression of the global signal may significantly change results when studying clinical populations, the global signal was preserved.

### DMN Identification

The group independent components analysis (ICA) method was used to pick out DMN components according to the templates provided by the Group ICA Of fMRI Toolbox (GIFT; http://mialab.mrn.org/software/gift) (34). Briefly, the ICA included primarily three steps performed in GIFT: data reduction, independent component separation, and back reconstruction. First, the optimal number of ICA components was set to 20, and subject- and group-level principal component analyses (PCAs) were performed to reduce their dimensions. Data from each subject were reduced using PCA according to certain components. The reduced data were separated by ICA using the extended informal algorithm. The group-level PCA was applied to further reduce the temporal dimension of the group fMRI data. The number of independent components (ICs) and time courses for each subject were retroactively reconstructed, and the mean spatial maps for each group were transformed to Z-scores for display (35, 36). Finally, the independent component that best matched the DMN as previous templates provided by GIFT was selected. The generated DMN was used as a mask for further NH analyses. We used voxel-wise one-sample *t*-test to set a statistical map and a threshold. According to the Gaussian random field (GRF) theory, *p*-value < 0.01 represents a significant statistical modification of multiple comparisons. Voxel significance and cluster significance values meet requirements at values of *p* < 0.01. The study created masks for the parts included in the DMN. The generated DMN was used as a mask for further NH analyses (8).

### NH Analysis

NH analysis was calculated by using a script in Matlab (Mathworks), using a process described previously (35). For each subject, we computed the correlation coefficients of each voxel against all other voxels within the DMN mask. Then, the mean correlation coefficients were averaged and subsequently changed into z value by using z-transformation. The resultant values generated the NH maps.

### Statistical Analyses

Demographic information, including age, sex, educational level, and imaging data, were compared between the MCI and the control groups. Categorical data were compared by χ^2^ test, and continuous variables were compared by the two-sample *t*-test. The NH maps of patients and healthy controls were analyzed with a two-sample *t*-test via voxel-wise cross-subject statistics within the DMN mask. We computed gray matter volumes of every participant and used them as confounders. *P*-value < 0.01 was set as the significance level. GRF theory was adopted to correct multiple comparisons (voxel significance: *p* < 0.001, GRF cluster corrected significance: *p* < 0.01). The correlations between the NH value and ADAS-Cog in patients were performed using Pearson's correlation. Bonferroni was used to correct the significant level at *p*-value < 0.05.

## Results

### Demographics and Clinical Characteristics of the Subjects

In total, 38 patients with MCI (age range 60–80) and 69 health controls (age range 59–82) were enrolled in the study. No subjects were discarded during this analysis. No significant differences were found between the two groups by gender (χ^2^ test *p*-value = 0.63, χ^2^ = 0.23), age (*p*-value = 0.95, *t*-tests *t* = 0.69), and years of education (*t*-tests *p*-value = 0.09, *t* = 0.88) (Table 1). The clinical data as mean ADAS-Cog score in MCI was 12.02.

**Table 1:** Characteristics of the participants.

**Demographic data**	**Patients (*n* = 38)**	**HC (*n* = 69)**	***T* (or χ^2^)**	***P*-value**
Gender (male/female)	38 (22/18)	69 (34/35)	0.23	0.63^a^
Age (years)	69.16 ± 4.96	68.46 ± 4.97	0.69	0.95^b^
Years of education (years)	9.16 ± 2.95	9.74 ± 3.38	0.88	0.09^b^
ADAS_Cog score	12.02 ± 1.58	–	–	–

a *The p-value for gender distribution was obtained by χ^2^ test*.

b *The p-value was obtained by two-sample t-tests*.

*HC, healthy control; ADAS_Cog, Alzheimer's disease assessment scale-Cognitive subscale*.

### The DMN Maps Determined by Group ICA

**Figure 1:**
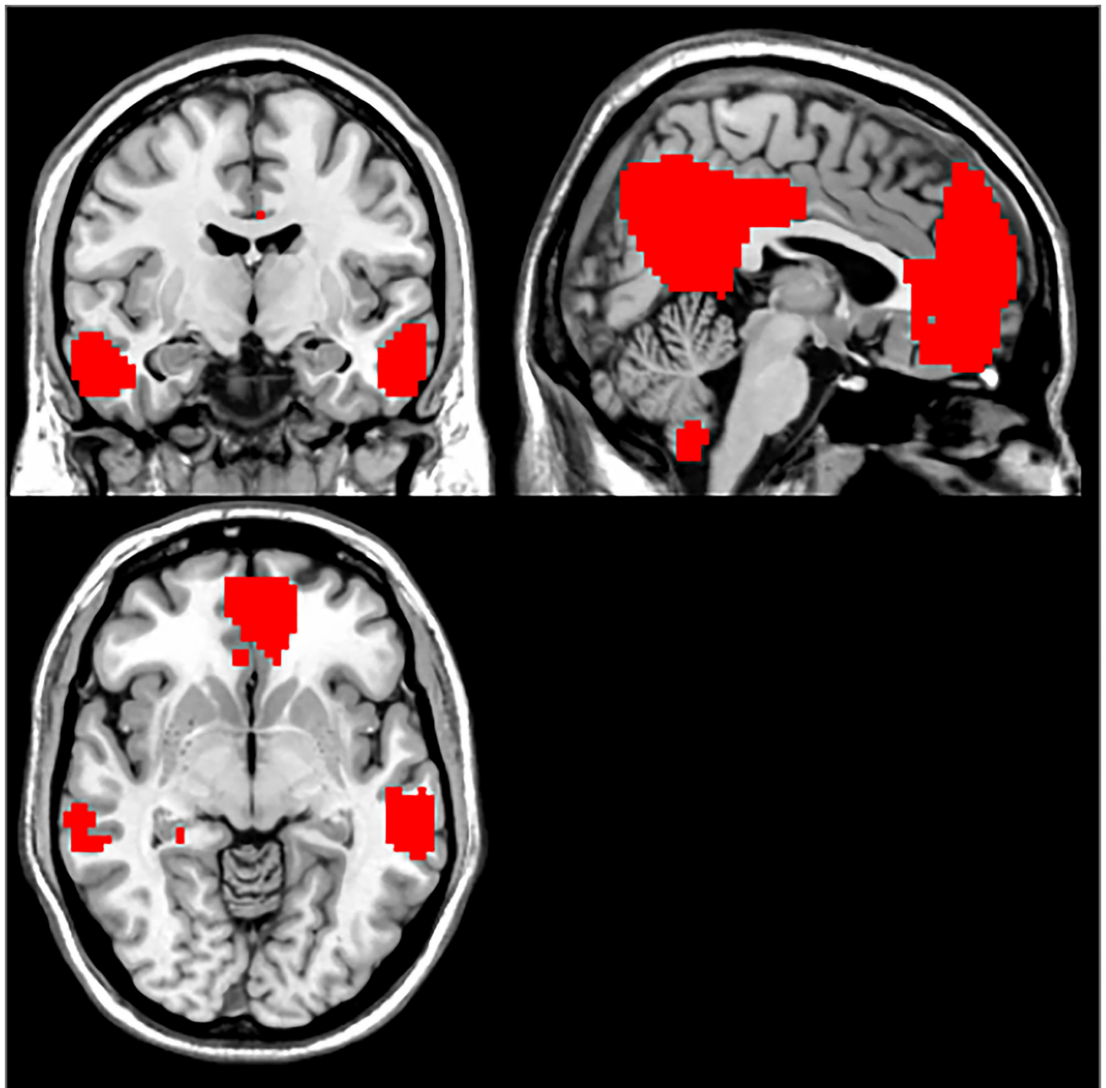
DMN mask.

The DMN was picked out from the control group as a mask by using the ICA method. The following brain regions were included in DMN: the bilateral medial prefrontal cortex (MPFC), the posterior cingulate cortex (PCC)/precuneus (PCu), ventral anterior cingulate cortex (ACC), lateral temporal cortex, medial, lateral, inferior parietal lobes, and cerebellum Crus 1 and Crus 2. The generated DMN mask was used in further NH analysis (Figure 1).

### NH: Group Differences in the DMN

**Figure 2:**
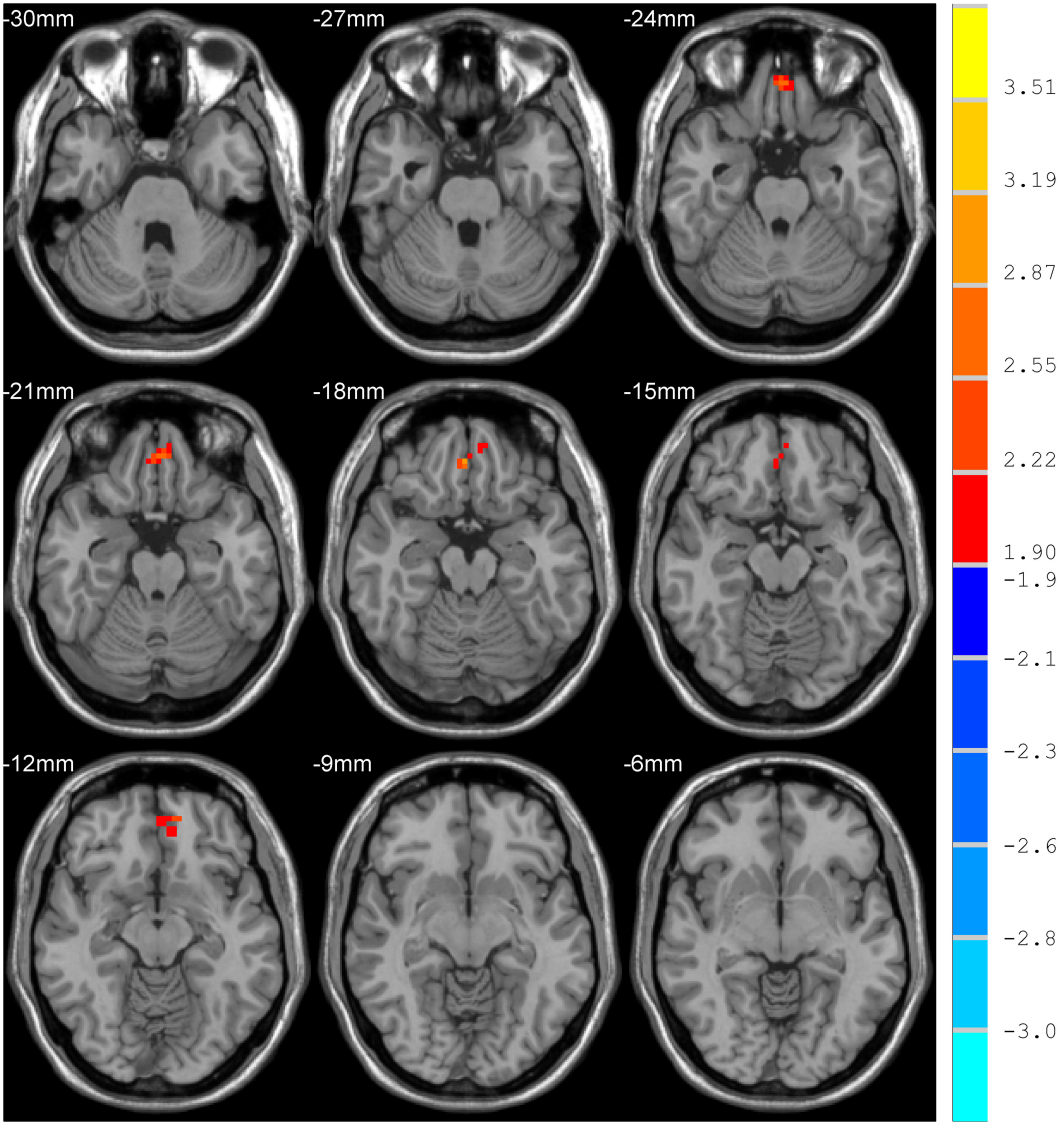
Increased NH in the ventral medial prefrontal cortex (vmPFC).

**Figure 3:**
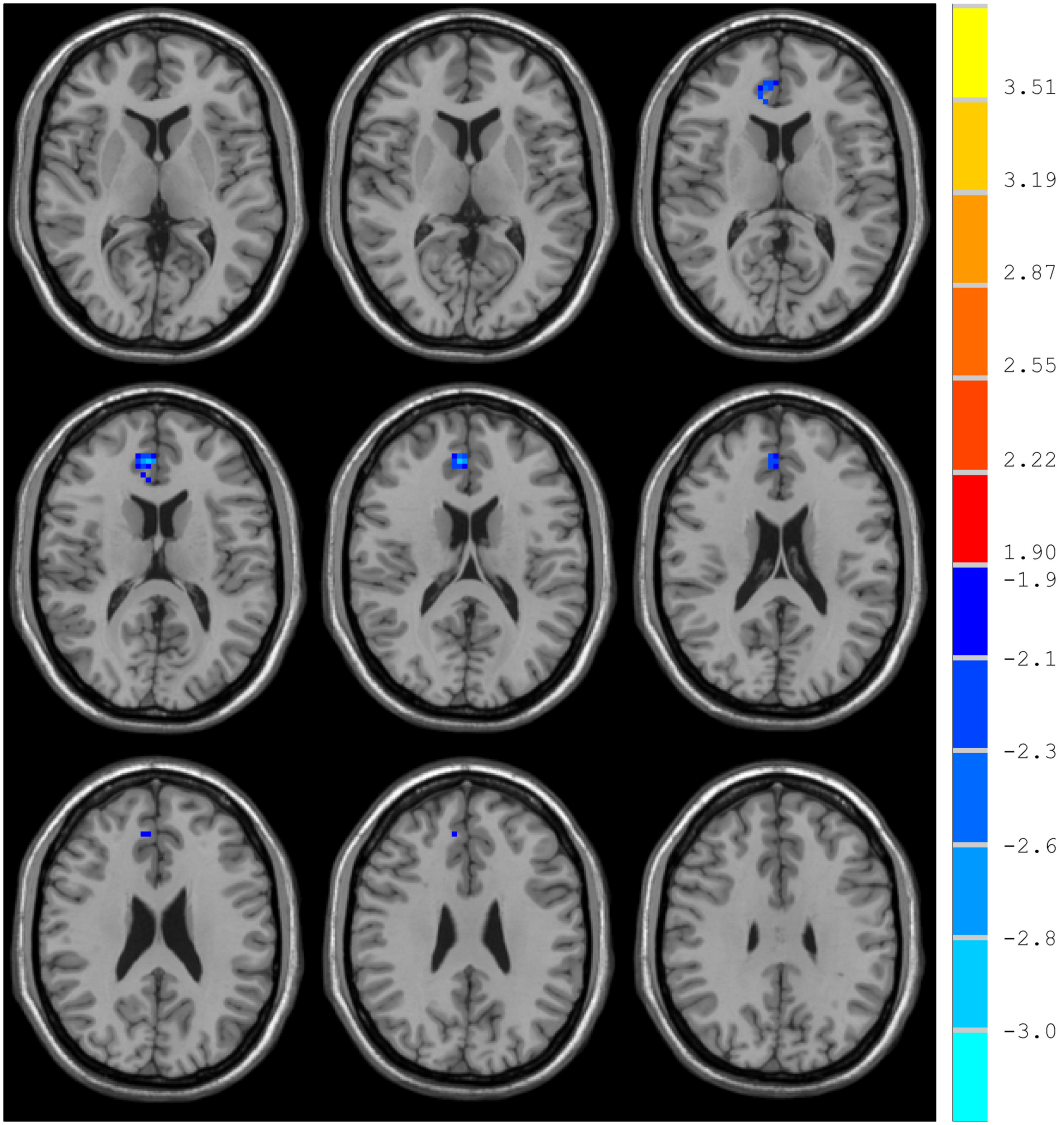
Decreased NH in the anterior cingulate cortex (ACC).

Compared with the control group, MCI patients showed decreased NH in the anterior cingulate cortex (ACC) and increased NH in the ventral medial prefrontal cortex (vmPFC) (Table 2, Figures 2, 3).

**Table 2:** Significant differences in NH values between the groups.

**Cluster location**	**Peak**	**(MNI)**		**Number of voxels**	***T*-value**
	**X**	**Y**	**Z**		
Patients < controls					
Right ACC	6	42	18	40	−2.93
Patients > controls					
Right vmPFC	3	42	−18	44	3.03

*MNI, Montreal Neurological Institute; ACC, anterior cingulate cortex; vmPFC, ventral medial prefrontal cortex*.

### Correlations Between NH and Clinical Variables

The mean NH values were extracted in the two regions (ACC and vmPFC), which showed significant group differences. In patient group, Pearson linear correlation analysis was performed to explore the correlation among NH and ADAS-Cog. No significant correlation was found between these NH values and any of the sub-scales of ADAS-Cog.

## Discussion

DMN is one of the most commonly involved resting-state networks in MCI research. Here, we provide the first comparison of NH of this network between MCI patients and age-matched controls. MCI patients showed decreased NH in the right ACC and increased NH in the vmPFC regions of the DMN.

The ACC is a critical region that appears to play a vital role in a wide variety of functions, not only in cognitive functions but also in diseases (15). Previous studies have exhibited that the functional ACC abnormalities are associated with cognitive impaired disorders (36, 37). Abnormal activity in the MPFC/ACC may lead to an alerting connection of the DMN and influence the suppression or activation in the DNM at task and rest (38). Thus, our results of lower NH in the right ACC may result in an inappropriate level of activity in the DMN and impede enough cognitive resources to be allocated into the cognitive function, which results in poor functional performance in MCI patients.

Our study showed increased NH in vmPFC, an essential node of the DMN (27). Previous research demonstrated similar findings, which also exhibited increased activity in bilateral vmPFC in the MCI using the method of ICA (28). However, contradictory results were found in some researches, which showed a decreased amplitude of low-frequency fluctuation in the right ventral vmPFC (29). Meanwhile, some structural investigations found a relationship between MCI and vmPFC. In the study of Zhao et al., MCI patients exhibited significant gray matter volume loss in vmPFC (30). The discrepancies of decreased and increased vmPFC activation in MCI may attribute to different methodological factors, such as the analytic method. They may also be due to differences in subjects, including sample size, symptom severity, and illness duration.

The DMN is intimately implicated in MCI, and the ACC and vmPFC are essential components of the DMN. In our study, mild cognition impaired patients exhibited lower NH values in the right ACC and higher NH values in the vmPFC than those of healthy controls. Previous ICA study showed decreased connectivity between the medial prefrontal cortex (mPFC) and ACC (25). Another seed-seed based analysis revealed decreased functional connectivity between the ACC and PCC in MCI subjects (39). Compared with methods used before to explore the DMN in MCI, such as ICA and seed-based correlation approach, NH has several potential advantages over existing approaches to examining resting-state functional connectivity. First, it allows for an unbiased survey of DMN and provides a straightforward method of assessing differences between groups without the need for the previous hypothesis of where abnormalities might be in the network (40).

Limitations of the study include the following. First, this study has a limited sample size. This might explain the lack of a significant correlation between abnormal NH values and clinical variables in the patients. Second, we focused on the abnormal NH in the DMN, which was primarily used to clarify the function of the DMN during the pathophysiology of MCI; in doing so, abnormal NH in other brain regions may have been neglected. Moreover, other potential confounding effects may be related to differences in vascular signals, especially in fMRI studies of neurodegenerative diseases (41). Generative models may help distinguish age and disease effects on the true neural connectivity from coupled neurovascular alterations (42–44).

In conclusion, this study aimed to investigate the NH of DMN in MCI rs-fMRI. Also, the present findings indicate the abnormal NH values in the DMN exist in MCI patients, which provides insight into the correlation between dysconnectivity in the DMN and cognitive deficits. Thus, the current results figure out the importance of the DMN regarding the pathophysiology of MCI.

## Data Availability Statement

The raw data supporting the conclusions of this article will be made available by the authors, without undue reservation.

## Ethics Statement

The studies involving human participants were reviewed and approved by Medical Ethics Committee of Wuxi Mental Health Center. The patients/participants provided their written informed consent to participate in this study.

## Author Contributions

XZ designed and supervised the study. ZC supervised the study. ZZ collected the original data. HY and YC managed and analyzed the data and wrote the manuscript. All authors contributed to the article and approved the submitted version.

## Conflict of Interest

The authors declare that the research was conducted in the absence of any commercial or financial relationships that could be construed as a potential conflict of interest.
